# Opening clinical encounters in an adult musculoskeletal setting

**DOI:** 10.1016/j.math.2014.03.011

**Published:** 2014-08

**Authors:** Emily C. Chester, Natalie C. Robinson, Lisa C. Roberts

**Affiliations:** a Faculty of Health Sciences, Building 45, University of Southampton, Highfield, Southampton, Hampshire, SO17 1BJ, United Kingdom; b Therapy Services Department, University Hospital Southampton NHS Foundation Trust, Tremona Road, Southampton, Hampshire, SO16 6YD, United Kingdom

**Keywords:** Communication, Interaction, History-taking, Musculoskeletal, Physiotherapy

## Abstract

Effective communication between healthcare professionals and their patients is crucial for successful consultations, and can profoundly affect patients' adherence to treatment. Despite this evidence, communication within the physiotherapy profession is still underexplored, in particular, how ‘best’ to open clinical encounters. This study explores the issue by seeking the preferences of physiotherapists for opening encounters in the adult musculoskeletal outpatient setting. Initially, 42 consultations and 17 first follow-up encounters were observed between qualified physiotherapists and patients with back pain. These encounters were audio-recorded, analysed and used to develop a questionnaire to determine clinicians' preferences for opening encounters. From these findings, a synopsis of the questionnaire was posted on the four most-relevant professional networks of the national, interactive Chartered Society of Physiotherapy (iCSP) website, to canvass opinion more widely. Among the 43 physiotherapists who responded, the preferred ‘key clinical question’ for an initial encounter was: “*Do you want to just tell me a little bit about [your ‘problem presentation’] first of all?*”; and for follow-up encounters: *‘How have you been since I last saw you?*’ These results provide an important and novel contribution to the profession, as debate on this issue has not previously been published. Although the sample size in this study is small, the aim of this paper is to generate reflection and debate among clinicians on their preferences for opening patient encounters and optimising the non-specific treatment effects.

## 1 Introduction

Effective communication between healthcare professionals and patients is crucial for a successful clinical encounter (Gask and Usherwood, 2002) and impacts upon every patient contact. A proficient ‘medical interview’ is fundamental to creating a good interpersonal relationship, as well as information exchange and optimal professional decision-making (Ong et al., 1995; Stewart, 1995). Furthermore, in developing patient-centred care, clinicians are advised to attend not only to the disease, but to the patient's experience of symptoms, the impact of the condition and what really matters for the patient (Pollock, 2001; Walseth et al., 2011).

It is imperative that healthcare professionals consider their communication skills right from the outset, as it is reported to take only 39 ms for a first impression to be made (Bar et al., 2006) and ‘many encounters’ to change (Tongue, 2007). The early stages of the clinical encounter are also when patients present their problems to the clinician. Heritage and Robinson (2006) introduced the term ‘problem presentation’ to describe the stage at which patients disclose information about their symptoms to the clinician. This important component is reported to be the only time in a medical encounter that patients are given the opportunity to describe their condition in their own words and address their own personal agenda (Heritage and Robinson, 2006).

When patients are given the opportunity to participate, they are more likely to work alongside the healthcare professional and have increased satisfaction with the outcome (Glueckauf, 1993; Payton et al., 1998), with both parties sharing knowledge and power (Edwards et al., 2004). However, research has also highlighted that clinicians' communication, and in particular, how they phrase their questions about the ‘problem presentation’, can affect patient ‘satisfaction’ (Heritage and Robinson, 2006; Robinson and Heritage, 2006) as well as adherence to treatment (Zolnierek and Dimatteo, 2009). Therefore, the clinician's communication skills are vital in establishing a good interpersonal relationship with patients, creating a welcoming environment, and enabling patients to freely express their issues.

To date, research into how clinicians “should” open their clinical encounters is at an early stage of development and predominantly focuses on physicians working in primary care settings (Heritage and Robinson, 2006; Robinson and Heritage, 2006). These studies explored opening encounters using video-recorded data; however it has been reported that a camera can alter the natural flow of communication between clinicians and patients (Roberts and Bucksey, 2007). Furthermore, it is unknown how well the findings from medical encounters translate to clinical encounters involving other healthcare professions.

Within physiotherapy, communication studies have tended to focus on, interactions and relationships, the content of encounters and the impact upon outcome (Roberts et al., 2013), with none specifically addressing the issue of opening clinical encounters. Therefore, this study aims to address the gap using a series of audio-recorded consultations from an outpatient setting, to identify the phrasing preferred by physiotherapists when opening clinical encounters in an adult musculoskeletal outpatient setting.

## 2 Method

### 2.1 Stage 1: observational study

#### 2.1.1 Study design

The first stage was a cross-sectional, observational study of interactions between musculoskeletal physiotherapists and patients with low back pain.

#### 2.1.2 Setting

This study took place in a primary care service in Southern England. Patients were referred to the service by their General Practitioner (GP), and were allocated an initial 45-min appointment with a physiotherapist, and further 30-min treatment sessions, as necessary.

#### 2.1.3 Participants

*Patients:* The patient sample (*n* = 42) comprised adults aged ≥18 years, referred with a diagnosis of low back pain (of unspecified duration), defined as pain in an area bounded by the 12th thoracic vertebra and ribs superiorly, gluteal folds inferiorly and contours of the trunk laterally. Patients with a history of recurrent back pain were included, provided they had received no physiotherapy/acupuncture within the preceding three months, in order to identify this episode of back pain as distinct.

The exclusion criteria were: signs and symptoms suggesting possible serious spinal pathology (red flags); spinal surgery for this episode; another musculoskeletal disorder more troublesome than the back pain; consultations (for this episode) with other health care professionals (excluding the GP); a known severe psychiatric or psychological disorder; and people who were unable to communicate in English without assistance.

*Clinicians*: All physiotherapists working in the study setting (*n* = 15), registered with the Health and Care Professions Council and currently managing patients with back pain, were invited to take part.

#### 2.1.4 Data collection

A small, digital Edirol audio-recorder (model R-09HR, Roland Corporation, Japan) was placed in the treatment cubicle. The senior researcher (LR) discreetly sat out of the direct field of vision of either participant and took no active part in the consultation, recording field notes to contextualise the events that took place during the encounter. The audio-recordings were transcribed verbatim and thematically analysed using a Framework approach (Ritchie et al., 2003).

#### 2.1.5 Findings from stage 1

From the 42 initial physiotherapy consultations, 11 key clinical questions were identified, which are summarised in Table 1 (column 3). From the 17 first follow-up encounters, 7 key clinical questions were identified, summarised in Table 2 (column 3). The wording of these questions was then used as the base for a national survey to determine clinicians' preferences.

### 2.2 Stage 2: survey

#### 2.2.1 Study design

A cross-sectional survey was carried out within the United Kingdom to identify how physiotherapists prefer to open their clinical encounters.

#### 2.2.2 Questionnaire

At the inception of the study, no appropriate measuring tool existed for determining preferences for opening clinical encounters. Therefore, a bespoke questionnaire was designed based on audio-recorded clinical encounters from stage one. The 42 initial consultations were thematically coded and the exact wording of the ‘key clinical question’ (KCQ) was identified, i.e. the first question where the physiotherapist asks the patient about their presenting problem, in this case, back pain. Topics discussed prior to this point (in the opening phase of the consultation) were also identified and collated. The exact phrasings of the KCQ were used in the questionnaire to optimise face and content validity.

The questionnaire was established electronically using QuestionMark Perception software and consisted of ten demographic and eight core questions, charting the initial consultation (four questions) and follow-up clinical encounter (four questions).

For the initial consultation, participants were asked to identify and rank their top five preferred phrasings for the KCQ out of the eleven options from stage one. They were also given an opportunity to identify any alternative phrasing of the KCQ they believed to be more effective or preferred from their own clinical practice. A similar format of questions was used for follow-up clinical encounters.

Prior to the main study, pilot work was conducted using a convenient sample of seven MSc physiotherapy students and five senior physiotherapists, to evaluate the questionnaire's acceptability and give participants the opportunity to comment on the layout, design and content of the questionnaire. Minor formatting changes were made to the questionnaire following this feedback.

#### 2.2.3 Data collection

Participants were recruited using the national, interactive Chartered Society of Physiotherapy website (iCSP). A synopsis of the study was included in the networks' fortnightly email bulletins of the four most relevant professional networks: i) Sports Medicine; ii) Orthopaedics; iii) Massage and Soft Tissue Therapy and; iv) Pain Management. At the time of recruitment, membership of the four networks totalled 34,922 (including possible duplicates). Members who were interested in the study were asked to contact the authors via email and were then sent a link to the electronic questionnaire and a participant information sheet. The sample included all available members of the four networks. In addition, the senior researcher (LR) publicised the study to delegates in a keynote address at the Physiotherapy Research Society (Sheffield, 2012). Data were collected between August and October 2012, and were coded for anonymity. One follow-up reminder was sent.

#### 2.2.4 Data analysis

Descriptive statistics were used to determine physiotherapists' preferred phrasing when opening clinical encounters.

Frequencies were reported for the topics clinicians discussed before or after their KCQ in both initial and follow-up clinical encounters, and a scoring system was used to determine the preferred phrasing. Each first choice phrase received a score of five; second choice received a score of four etc.; down to the fifth choice, which received a score of one. These scores were then summed for each phrase, to identify the most popular.

Data were managed using SPSS for Microsoft Windows, Release 20.0 (IBM: SPSS Inc) and Microsoft Excel 2010.

Ethical approval was granted from the Faculty of Health Sciences, University of Southampton.

## 3 Results of national survey

### 3.1 Demographics

A total of forty-three participants responded, however three questionnaires were incomplete. The sample included qualified physiotherapists (*n* = 31) and students (*n* = 12), of whom 18 (42%) were male and 25 (58%) female. The number of years experience in musculoskeletal physiotherapy ranged from 2 months to 29 years (mean: 10 years 10 months, median: 10 years 2 months) and the number of years qualified ranged from 1 year 3 months–37 years (mean: 13 years 10 months, median: 12 years 2 months).

#### 3.1.1 Initial clinical encounter

##### 3.1.1.1 Pre-key clinical question topics

The majority of respondents reported including the following topics in their clinical encounter before raising the KCQ: i) a general greeting (*n* = 39); ii) an introduction of their name (*n* = 38) and role (*n* = 31); iii) an explanation of what would be involved in the consultation (*n* = 31); iv) confirmation of referrer details (*n* = 28); and v) a check of the patient's personal details (*n* = 32), and preferred name (*n* = 33). Additionally, 16% (*n* = 7) reported mentioning parking and directions, and 30% (*n* = 13) the weather.

##### 3.1.1.2 Key clinical question

The preferred phrasing of the KCQ in an initial clinical encounter was *“Do you want to just tell me a little bit about (your ‘problem presentation’) first of all?”* (score: 83). Preferences for the KCQs are summarised in Table 1.

When clinicians were asked for their own preference for opening a clinical encounter (i.e. not from the audio-recordings), a shared theme that arose was to explicitly ask about the patients' presenting problems and why they had come to physiotherapy in their own words.

The themes participants identified as ‘missing’ from the questionnaire included: a check to see if patients had seen a physiotherapist before; establishing whether patients understood why they had been referred; and their understanding of the role of physiotherapy.

#### 3.1.2 Follow-up clinical encounter

##### 3.1.2.1 Pre-key clinical question topics

In the follow-up consultations, clinicians reported greeting the patient (*n* = 38), giving a summary of the previous clinical encounters findings (*n* = 20), and explaining what would be involved in the follow-up consultation (*n* = 20) prior to asking them about their problem presentation. Additionally, 14% (*n* = 6) of respondents reported mentioning parking, 5% (*n* = 2) directions and 37% (*n* = 16) weather, before the KCQ. An additional topic respondents deemed important to bring up was to check how the patient felt after their initial physiotherapy session.

##### 3.1.2.2 Key clinical question

The preferred phrasing of the KCQ by physiotherapists in a follow-up clinical encounter was *“How have you been since I last saw you?”* (score: 158). Preferences of KCQs in the follow-up encounters are summarised in Table 2. When asked if they had any other preferred ways of opening the encounters, a theme emerged of asking directly about the patient's symptoms.

#### 3.1.3 Audio-recorded consultation/clinical encounters

From the 42 audio-recorded initial consultations, 19% (*n* = 8) of the KCQs were open, 17% (*n* = 7) were open-focused and 64% (*n* = 27) were closed. Open questions elicited on average a 22.6 s (s) (range 1–49 s) response from the patient about their presenting problem, whilst open-focused and closed questions elicited on average 47.1 s (range 8–69 s) and 15.4 s (range 1–90 s) responses, respectively. When answering the KCQ, patients were interrupted by the physiotherapist in 25 out of 42 consultations (60%), whereas in the other 17 consultations (40%), patients' answers came to a natural stop before the physiotherapist spoke. Out of these 25 interrupted consultations, responses to closed questions (*n* = 16) were interrupted sooner (mean = 19.9 s) than open (*n* = 4) (mean = 24.8 s) and open-focused questions (*n* = 5) (mean = 45.2 s).

## 4 Discussion

This exploratory study aimed to identify the preferred phrasing of physiotherapists when opening clinical encounters in musculoskeletal outpatient settings. The results indicate that clinicians are in favour of using open questions when asking patients about their *‘*problem presentation’ in both initial and follow-up clinical encounters. Open questions give patients the opportunity to express their own ideas and experiences freely, whereas closed questions only look for a ‘yes’, ‘no’ or simple fact response (Evans et al., 2008). These results relate to previous research, which has highlighted that when practitioners use open questions at the start of their consultations, patients report greater satisfaction and adherence to treatment, as they feel the practitioner has listened to them, which facilitates the therapeutic relationship (Robinson and Heritage, 2006; Zolnierek and Dimatteo, 2009).

In the present study, physiotherapists favoured the question: “*Do you just want to tell me a little bit about [your problem presentation] first of all?*” which is a problem-focused symptom query, and is both a question and an invitation. In lay terms, this could be described as an open-focused question, as it allows the patient to direct the problem aspect. The ‘just … tell me’ component is eliciting a narrative and the clinician is not presupposing a specific angle or problem, or displaying prior knowledge of the patient's problem, thereby occupying a less knowledgeable (K–) epistemic status (Heritage, 2012). It is evident that the question is phrased as a yes/no interrogative and displays an entitlement contingency, however it still gives the patient an entitlement to decline. Finally, the temporal component ‘first of all’ sets up that there is more to come and the patient will therefore have further opportunities to tell their story. This style of question was favoured over:

• narrative open questions (“*Do you want to tell me your story*”);
• questions that focus on a single, specific issue (“*Do you want to start off by telling me whereabouts you’re getting your pain at the moment?*”);
• closed questions (“*The referral says you’ve got [‘problem presentation’] is this correct?*”)

The findings of the current study are comparable to that of Marvel et al. (1999), who observed that in 34 out of 264 interviews between physicians and patients, physicians followed open questions with open-focused questions when addressing patients' agendas. They commented that this is a ‘useful’ style to adopt as it avoids gathering an extensive list of patient concerns rigidly at the opening of the interview (Marvel et al., 1999). Therefore, asking a question that allows the patient to express their problem in their own words, but gives them a focus, may deter the patient from deviating from the topic and also help to achieve two of the most important communication skills in a therapeutic setting: the ability to truly hear what the patient is trying to say; and the ability to allow the patient to speak without interruption (Jackson, 2006).

Interestingly, in the current study, subsequent analysis of the 42 audio-recorded initial consultations demonstrated that physiotherapists were inclined to interrupt the patient when answering the KCQ in 60% of cases. Marvel et al. (1999) also found that 45.5% of patients were interrupted while giving their ‘statement of concerns’ and this was associated with fewer concerns mentioned by patients, late-arising concerns, and missed opportunities to gather important patient data. On average, patients were given 23.1 s to itemise their concerns before interruption from the physician (Marvel et al., 1999), although the study did not address the type of questions that were used prior to the patients' response and whether these questions may have influenced the response time. Analysis of the audio-recorded encounters in the current study revealed that physiotherapists allowed patients to speak for the longest without interruption following open-focused questions. This could be because physiotherapists were gleaning useful information, and patients felt comfortable to express their concerns in their own words.

Clinicians are encouraged not to interrupt patients' opening statements because determining reasons for the patient pursuing professional help is important to a successful clinical encounter (Beckman and Frankel, 1984) and it is reported there is an increased ‘fondness’ towards the clinician as the patient discloses this health information (Collins and Miller, 1994). Patients will typically take 92 s to explain their ‘problem presentation’ in an outpatient setting if not interrupted (Langewitz et al., 2002) and will present with an all-round summary of their problems (Walter et al., 2005). Yet, none of the patients in the 42 initial encounters in the current study spoke for 92 s, whether given this opportunity or not.

In addition to the phrasing of the key clinical question, establishing a relationship with the patient at the outset of the consultation, has been considered to be an essential element of physician-patient communication (Makoul, 2001). This ‘opening phase’, where the healthcare professional has greeted the patient and familiarised themselves with the patient's records is important, as it has the potential to influence the length of the patient's response about their ‘problem presentation’, depending on how comfortable they are made to feel by the clinician (Coupland et al., 1994; Robinson, 1998). The physiotherapists in the present study built rapport in the ‘opening phase’ by reportedly greeting their patients, introducing themselves and their role, explaining the physiotherapy consultation, asking patients about personal details, and confirming the referral details.

It is interesting to note however, that the initial topics raised mainly related to the physiotherapist gathering and giving information, and did not allow the patient to engage in ‘small talk’. For example, only a minority of physiotherapists brought up topics such as the weather, parking and directions. This correlates with the finding of Roberts and Bucksey (2007) that physiotherapists make approximately twice as many statements as patients, and the verbal communication used by physiotherapists comprises mostly ‘content behaviors’, such as taking history and giving advice. Although ‘small talk’ has previously been described as a means of patients and physicians exhibiting ‘disattentiveness’ in medical interactions (Maynard and Hudak, 2008), in contrast, it has been attested that ‘small talk’ can help establish relationships because of its ability to ‘oil the social wheels’ of discourse (Holmes, 2000, p57), and thus facilitate instrumental behaviours within the consultation, such as willingness to disclose relevant health-related information (Hudak and Maynard, 2011).

### 4.1 Clinical implications

Professional and regulatory bodies pertinent to physiotherapy, recognise the importance of developing effective communication (HCPC, 2012; CSP, 2012). Therefore, knowing how clinicians and patients communicate, and specifically, how clinical encounters are “best” opened, is important for teaching and feedback to assist clinicians in optimising their non-specific treatment effects.

Parry and Brown (2009) recommend that teaching on communication at pre-qualification level should be based on existing empirical knowledge, but there are significant gaps in the evidence, which pose challenges for educators, students and researchers in this field. The contribution of the current study is that: i) educators in the field should consider the use of open-focused questions when advising about opening clinical encounters and; ii) clinicians could use these types of questions to facilitate patient engagement.

### 4.2 Limitations

Although this study is novel in researching physiotherapists' preferences on how to open clinical encounters, some limitations exist, in particular, the low response rate due to an ineffective recruitment strategy. This was hindered by being unable to put a direct link to the questionnaire on the iCSP website (due to ethical constraints). Therefore, participants had to email the researchers to request a link to the questionnaire, which subsequently may have deterred iCSP members from participating in the study.

Furthermore, this study only considered verbal communication between the physiotherapist and their patient, despite the recognition that communication relies on non-verbal as well as verbal communication (Hall and Lloyd, 1990; Oliver and Redfern, 1991; Caris-Verhallen et al., 1999; Waddell, 2004, p. 243; HCPC, 2012). Further research is required to investigate the impact of non-verbal and verbal communication skills and further work is currently in progress to address this from the patients' perspective.

## 5 Conclusions and recommendations

Effective communication is essential in healthcare and it has been reported previously that a clinician's choice of questioning can influence patients' responses and the subsequent outcome of the encounter. This study demonstrates that physiotherapists prefer open-focused questions when addressing the topic of patients' presenting problems in initial clinical encounters, providing patients with a focus, whilst still allowing them to express themselves in their own words. Furthermore, the study has highlighted that physiotherapists are inclined to interrupt patients as they respond to the key clinical question in 60% of encounters, which may negate this opportunity for patients to express what really matters to them. Further research is currently underway to explore this.

These findings should be interpreted with caution, due to the small sample size of the study. Nonetheless, they are a snapshot of physiotherapists' opinions and a foundation for future research. Considering the integral role that communication plays in every clinical encounter, it is suggested that more robust empirical evidence on opening encounters needs to be provided for the physiotherapy profession, including patients' preferences and the impact on outcome. In the current healthcare systems, it is vital that clinicians make every effort to maximise their non-specific treatment effects and enhance outcomes.

## Figures and Tables

**Table 1:** Results from the national survey showing the preferred phrasing of the key clinical question in initial consultations between physiotherapists and patients with low back pain.

Preferences	Score	Phrase
1st	83	Do you want to just tell me a little bit about [problem presentation] first of all?
2nd	77	I've had this referral through. Tell me what's happened.
3rd	71	The referral says you've got [problem presentation] is this correct?
4th	65	How can I help you today?
5th	57	What we'll do today is just have a bit of a chat about [problem presentation], I believe it is. All right?
6th	45	It's your [problem presentation e.g. knee, back etc.] that you're here for is it?
7th	35	What problem are you having at the moment?
8th	30	Do you want to tell me your story?
9th	29	Do you want to start off by telling me whereabouts you're getting your pain at the moment?
10th	28	I know a little bit from the GP, when did this start?
11th	17	How long have you had [problem presentation] for?

**Table 2:** Results from the national survey showing the preferred phrasing of the key clinical question in a follow-up clinical encounter between physiotherapists and patients with low back pain.

Preferences	Score	Phrase
1st	158	How have you been since I last saw you?
2nd	131	How did you get on with the [treatment e.g. exercises, hydro, injection, massage]?
3rd	82	How have you been feeling from a [problem presentation e.g. knee, back etc.] point of view?
4th	71	How are you getting on?
5th	54	How have you been?
6th	18	Are the [problem presentation e.g. knee, back etc.] symptoms on-going?
7th	12	What was the take home message that you got from me last time?
